# Fighting Back against Antimicrobial Resistance with Comprehensive Policy and Education: A Narrative Review

**DOI:** 10.3390/antibiotics11050644

**Published:** 2022-05-11

**Authors:** Justin F. Hayes

**Affiliations:** Department of Medicine, Division of Infectious Diseases, Banner University Medical Center-Tucson and South, University of Arizona College of Medicine, 1501 N. Campbell Avenue, Tucson, AZ 85724, USA; justinhayes@email.arizona.edu

**Keywords:** antimicrobial resistance, antimicrobial stewardship, public health, global health, drug development, environmental health, one health, medical education, curriculum

## Abstract

Globally, antimicrobial resistance has emerged as a significant threat. A comprehensive plan is required to combat antimicrobial resistance. There have been national and international efforts to address this global health problem, but much work remains. Enhanced funding and regulations to support antimicrobial stewardship policy and program development, reforms to incentivize drug development to treat resistant pathogens, and efforts to strengthen One Health programs are areas for collaboration and innovation. Finally, implementation of educational interventions for trainees encompassing these key areas along with training on policy and leadership development is critical to enable sustainability of these efforts to fight back against antimicrobial resistance.

## 1. Introduction

Antimicrobial resistance is a pressing threat. The Centers for Disease Control and Prevention (CDC) reported in 2019 that 2.8 million infections and 32,000 deaths due to resistant pathogens occur each year [1]. In addition, a comprehensive global analysis that included 23 pathogens and 88 pathogen-drug combinations from over 200 countries and territories was undertaken to estimate the overall burden of antimicrobial resistance in 2019. The analysis revealed that bacterial resistance was responsible for an estimated 4.95 million deaths, with 1.27 million deaths directly attributable to bacterial antimicrobial resistance [2]. Additionally, the World Health Organization (WHO) has endorsed guidance to monitor and evaluate rising antimicrobial resistance and delegated responsibilities for country governments, the One Health Tripartite mission, and other global partners [3]. 

In this review, the threat of rising antimicrobial resistance and the global response, antimicrobial stewardship policy and program development, drug development initiatives targeted to resistant pathogens, and environmental, agricultural, and One Health policy are described. Finally, the importance of the incorporation of policy related to antimicrobial resistance into medical and veterinary education is discussed.

## 2. The Threat of Antimicrobial Resistance and the Global Response

To highlight the urgent threats posed by antimicrobial resistance, the CDC has produced a report (updated in 2019) to characterize the significant issues related to antimicrobial resistance as well threat level designations for resistant pathogens [1]. The pathogens are divided into three categories: urgent, serious, and concerning. The urgent threats are carbapenem-resistant *Acinetobacter*, *Candida auris*, *Clostridioides difficile*, Carbapenem-resistant Enterobacteriaceae, and drug-resistant *Neisseria gonorrhoeae*, which are discussed further below.

Carbapenem-resistant *Acinetobacter* has a propensity to survive for long periods of time on surfaces and is usually found in individuals who have recently received care in a healthcare facility [4]. Drug-resistant *Candida auris* has emerged as a significant threat due to its ability to cause severe infections and easily spread in healthcare facilities, particularly long-term care facilities [5]. It has also been reported to have the ability to produce resistance to all major classes of antifungal agents [6]. *Clostridioides difficile* is a life-threatening cause of diarrhea and is associated with antibiotic use and time spent in healthcare facilities. Reduction in antibiotic use and stringent infection prevention policy have significant benefits towards preventing this infection [7]. Carbapenem-resistant Enterobacteriaceae is a major threat due to its ability to produce resistance to the broadest class of antibiotics (i.e., carbapenems) [8]. Importantly, robust measures to detect these organisms and prevent the spread of this notable resistance have been enacted by public health agencies [1]. Finally, drug-resistant *Neisseria Gonorrhoeae* is a different type of threat in that this is a sexually transmitted infection (STI) that can result in life-threatening complications, such as ectopic pregnancy and infertility [9]. There is also a risk of spread of additional STIs from individuals infected with this organism. Over time, resistance development in *Neisseria Gonorrhoeae* has led to a reduction in treatment options [10].

With these urgent threats, policymakers and stakeholders have advanced several initiatives in this arena. For example, the National Action Plan for Combating Antibiotic-Resistant Bacteria (CARB) was part of a national response by the United States (US) government focused on addressing antimicrobial resistance and was launched as a pathway to address the threat of rising antimicrobial resistance through a coordinated and collaborative effort. Specifically, the action plan focused on five areas: (1) slowing and preventing the emergence of resistant bacteria, (2) strengthening One Health surveillance efforts, (3) advancing the development and use of rapid and novel diagnostics for detection of resistant organisms, (4) accelerating research for new antibiotics, alternative therapeutics, and vaccines, and (5) improving global collaboration [11]. In addition, government agencies have collaborated to promote innovation in this area through several actions, including the Antimicrobial Resistance Challenge, advancement of the mission at the 2016 United Nations General Assembly, and collaborations with the WHO [1].

In fact, the WHO has adopted an action plan focused on antimicrobial resistance that outlines five objectives: (1) improved awareness of antimicrobial resistance through effective communication and education, (2) strengthened knowledge and evidence base for surveillance and research, (3) reduction in the incidence of infection through infection prevention and hygiene measures, (4) optimization of usage of antimicrobials in both human and animal health, and (5) development of the economic case for sustainable investment taking into account the needs of all countries [12].

From a public health standpoint, the CDC has led a multifaceted effort. This includes initiatives designed to not only detect and treat resistance in a timely fashion but also invest in prevention measures. Specific CDC actions include development of an Antibiotic Resistance Lab Network [13], the Antibiotic Resistance Solutions Initiative [14], and core guidance for antimicrobial stewardship in many health care settings [15]. Going forward, a robust public health infrastructure to promote optimal surveillance is critical.

In addition to these efforts and actions, the public’s understanding of the threat of antimicrobial resistance is critically important. A survey analysis conducted with the use of the Amazon Mechanical Turk Crowdsourcing platform to recruit participants demonstrated that although a substantial majority of respondents (93%) noted that inappropriate antibiotic use contributes to antibiotic resistance, 70% of survey participants responded neutral or disagreed with the statement that antibiotic resistance is a problem [16]. In another poll, 65% of Americans felt antibiotic resistance is a public health problem, while 81% were concerned that infections will become more difficult to treat due to antibiotic resistance [17]. As part of the CDC’s efforts to combat antimicrobial resistance and engage the public, an annual observance for raising awareness about the threat of antimicrobial resistance is conducted [18]. Increased educational events geared towards the public about the significant burden antimicrobial-resistant infections have on healthcare resources and the collective issues involved in a comprehensive approach to fighting antimicrobial resistance will remain important going forward.

## 3. Antimicrobial Stewardship

Antimicrobial stewardship programs (ASPs) are instrumental and pivotal in the fight against antimicrobial resistance. ASPs are effective at providing high-value care by reducing unnecessary antimicrobials, reducing rates of antimicrobial resistance, reducing healthcare-associated infections, and reducing costs [19,20]. In addition, ASPs are perfectly designed to enhance the critical areas of patient safety and quality as well as drive increased value in health care [21]. These programs work to promote antimicrobial stewardship with a primary goal “to optimize clinical outcomes while minimizing unintended consequences of antimicrobial use, including toxicity, the selection of pathogenic organisms (such as *Clostridium difficile*), and the emergence of resistance” [19].

The CDC has targeted antimicrobial stewardship by issuing guidance known as the Core Elements of Hospital Antibiotic Stewardship Programs [15]. The core elements are geared toward enabling hospitals of varying sizes and complexity to address the threat of antimicrobial resistance and promote patient safety with implementation of successful ASPs. The guidance takes note of the dynamic nature of antimicrobial stewardship and the need for increased flexibility in implementation of projects and programs. The core elements include leadership commitment, accountability, pharmacy expertise, action, tracking, reporting, and education (Table 1) [15].

The guidance has been a catalyst for federal policy and mandates. Effective 1 January 2017, The Joint Commission mandated that all hospitals have a formal ASP for accreditation [22], with much of the mandate including aspects of the CDC’s core elements for ASPs. Further support and engagement came in 2019 from the Centers for Medicare and Medicaid Services, which mandated the formation and development of an ASP as a condition of participation for all acute care hospitals that participate in Medicare and Medicaid programs [23]. Over the years, these policies and actions have encouraged further development and implementation of ASPs in other settings, including integrated and non-integrated health systems. In fact, innovative health systems have begun to promote and fund ASPs.

Atrium Health developed an ASP collaborative across their entire health system with enhanced funding for a centralized advisory ASP team that included representatives from pharmacy, infectious diseases, and data analytics [24]. At that time, Atrium Health consisted of 28 acute care facilities located in North Carolina, South Carolina, and Georgia. The mix was diverse with a structure consisting of small and critical access hospitals to larger academic medical centers. Initially, an advisory team of experts was assembled, and an analysis of the baseline state of ASPs across the entire network was performed. A system-wide antibiotic reduction goal depending on the maturity of each facility’s ASP was implemented with enhanced education and on-the-ground site visits. Several themes were acknowledged throughout the implementation of the ASP across the continuum, including communication, creation of more resources, optimizing technology, education, and design of specific antimicrobial stewardship targets. After implementation of these efforts, nearly all hospitals (25/28) reduced broad antimicrobial use and met the CDC core elements [24]. There were no data regarding other clinical outcomes, but this was difficult due to the variability and differences among all the hospitals in the system.

In another example, a nonprofit health system called Intermountain Healthcare specifically evaluated ASP implementation in 15 of its community hospitals [25]. Each of these hospitals contained less than 200 beds. A cluster-randomized design intervention was performed, and the hospitals were broken down into three program types (i.e., program 1, program 2, and program 3). Each program type had an escalating intensity of antimicrobial stewardship interventions, with program 3 being the most intense. All hospitals had access to a hotline with infectious diseases expertise and received monthly reports and education, but only program 3 had a widespread implementation of prospective audit and feedback as well as more restrictions placed on antibiotics than both programs 1 and 2 type hospitals. The results showed that only program 3 hospitals had a significant reduction in both total antibiotic use (11% decrease from baseline) and broad-spectrum antibiotic use (24% decrease from baseline). The use of restricted antibiotics did decline at all program-type hospitals but was again most significant in program 3 hospitals [25]. The study demonstrated the importance of implementing ASPs in smaller hospitals to provide enhanced high-value care and to reduce antimicrobial usage and expenditures. The study also demonstrated that ASPs require modification and escalating levels of intensity to reach more impressive reductions in antibiotic use as well as enhanced quality of care at a hospital. In addition, the study also provided an example of how to incorporate ASP interventions into the healthcare delivery apparatus of a larger system.

Finally, a statewide collaborative demonstrated another way to address and improve antimicrobial stewardship. The Michigan Hospital Medicine Safety Consortium is a statewide initiative consisting of hospitals across the state of Michigan [26]. The consortium was utilized to focus on antimicrobial stewardship. Survey methods were implemented to identify key areas of need to improve the existing and current ASPs at Michigan hospitals. The response rate was high at 100%, and nearly all hospitals already had an existing ASP. The results revealed that although most hospitals had programs in place targeted to specific infectious disease syndromes, monitoring of adherence to recommendations was lacking. Overall, four areas for improvement were identified: clinically relevant antibiotic use data, monitoring of compliance, syndrome-specific initiatives, and discharge antimicrobial stewardship [27]. The analysis demonstrated that merely having a specific quality initiative with some recommendations will not fully address critical issues such as antimicrobial stewardship. There can be a role for significant improvement of a health system through sharing of best practices, advancing quality initiatives, and successful collaboration. Continued efforts to promote policy and develop structured ASPs will be critical, including further development of ASPs in other settings, such as long-term care facilities and ambulatory practices.

## 4. Drug Development

To address the rise in antimicrobial resistance across the US and the world, drug development is crucial. This includes not only the development of antibacterials targeted to bacterial resistance [28,29,30,31] but also new antifungals that are less toxic and provide coverage against antifungal resistance as well as rare fungal infections [32,33,34,35]. Unfortunately, this effort has not had the success needed due to a variety of challenges. Larger pharmaceutical companies abandon this field due to more profitable areas in the industry. This is primarily based on the decisions that are involved in the investment in drug development. Specifically, the investment involves consideration of a product’s net present value, which is the projected earnings minus expected production costs [36]. In addition to net present value calculations, other issues have led to lack of success, including slow clinical uptake of newer antibiotics, a rarity of specific infections in the US that the new antibiotics are designed to target, and an overall lack of innovation [36].

For the development of new antibiotics directed toward resistant infections, traditional pharmaceutical investment in this field is a losing proposition. However, a group of pharmaceutical companies implemented the AMR Fund in July 2020 focused on bringing new antibiotics to the market by 2030 through the provision of pharmacy expertise and technical support along with fostering of public-private partnerships for sustainable drug development [37]. This has the capacity to leverage more resources for smaller companies that have a desire to invest in the field of drug development for antimicrobial-resistant infections.

In addition, there are several reforms that have been either implemented or discussed to help with the critical need for drug development for resistant pathogens. First, reimbursement reforms aim to encourage development in this field by increasing payments to hospitals that use novel antimicrobials active against antimicrobial resistance. Specifically, the Disarm Act is currently a policy under consideration in US Congress to further expand this area by directly impacting reimbursement to hospitals for the use of these new antibiotics if the hospitals have ASPs and report on various measures related to antimicrobial usage. [36,38] Second, subscription reforms are policies that allow for allocation of money from governments to pay companies with a guaranteed revenue per year to ensure access to antibiotics targeted for antimicrobial resistance and have been done in the United Kingdom. [36,39] In the US, the Pasteur Act was proposed as a subscription reform to incentivize work in this area by creating subscription contracts for critical need antimicrobials. [36,40] Finally, push incentives are types of programs that encourage and incentivize development and progress in this field by giving up-front payments from the government for investment in drug development against antimicrobial resistance with a specific area of focus on early-stage drug development and innovation [36]. In general, a review of the literature has not found a consensus as to the optimal strategy for antimicrobial drug development and whether a national or international strategy should be utilized [41].

Although expansion and reform of drug development directed towards antimicrobial-resistant infections are vital, alternative measures are also needed to move beyond drug development. These include addressing the rational use of antimicrobials (see Section 3), as well as the development of novel diagnostics, vaccines, and therapeutics.

Diagnostics development can enhance the possibility of diagnosis of infection and specific resistance mechanisms allowing for more optimal antibiotic therapy. Modern technology is expanding this field, which enables more effective antimicrobial decision-making [42].

Vaccine development helps to enhance the ability to focus on the prevention of targeted diseases, including antimicrobial-resistant infections. Robust work in the area of modern vaccinology may lead to serious advances in combating antimicrobial resistance [43].

In addition to the development of new diagnostics and vaccines, phage therapy has received attention as a novel therapeutic strategy to combat antimicrobial resistance. In a striking example, a 68-year-old man suffered from a serious infection due to multidrug-resistant *Acinetobacter baumanii* unresponsive to conventional antimicrobial therapy and source control attempts. Clinical isolates were cultured from multiple drains, peritoneal fluid, and respiratory secretions, and phages were selected for treatment. Intravenous and percutaneous administration of phages to the source of infection was associated with clinical improvement and clearance of the resistant bacteria [44]. The use of phage therapy has continued to expand through clinical trials and expanded access and compassionate use programs, but methodology and approaches to administration have varied widely. The Antibacterial Resistance Leadership Group (ARLG) convened a group of experts on phage therapy that developed guidance around important clinical questions regarding phage therapy, including potential clinical scenarios where phage therapy could be used, laboratory testing, and pharmacokinetic issues [45]. Future work in this area will be important to watch.

Finally, to fully address the problem of drug development for antimicrobial resistance, organization among the multiple components in the antibiotic delivery system is required. These include measures such as finance, regulation, technology, and evaluation of the balance between access and excess use of antimicrobials [46]. These aspects are complex and require additional collaborations.

## 5. Environment/Agriculture/One Health

The community and environment are closely connected and provide opportunities for spread of resistant organisms. Human activity can introduce antibiotics and antibiotic-resistant organisms into the environment through soil and water [47,48]. In addition, resistant organisms and infections can spread in the environment by way of international travel. This can be through humans, animals, contaminated food and water, as well as time spent in healthcare facilities [49,50]. In the health care setting, waste can contribute to the spread of resistant organisms, while untreated sewage in the community can further contribute to contamination in the environment [1]. Finally, use of antimicrobials as pesticides can result in further spread of resistance through the environment [51,52].

To combat the issues contributing to the spread of resistance in communities and health care settings, policy is critical. As an example, the American Veterinary Medical Association has established core principles related to antibiotic stewardship in animal settings by implementing and supporting policy and guidance to promote more judicious use of antimicrobials in animals [53]. In addition, The Food and Drug Administration’s Center for Veterinary Medicine has established policies for a thorough risk assessment of antimicrobial use in animals that considers the ability of antibiotics used in food-producing animals to contribute to the risk of antimicrobial resistance, enables systematic evaluation of drugs used on animals, and promotes evaluation of any drugs used in food-producing animals for residue or other potentially harmful effects on humans, including the intestinal flora [54].

Another related aspect connecting the environment to resistance development and spread is the use of growth promotion in animal feeds. This strategy involves low doses of prophylactic antibiotics for growth promotion in animals, but the effect of the prophylactic antibiotic use on the development of resistance remains a serious concern [55]. Specifically, the literature has highlighted the risk of resistance related to antibiotic use in animals on human health [56]. Europe has had a stricter policy on this issue compared to the US by placement of bans on prophylactic antibiotic use in animals. These bans have been placed on growth-promoting antibiotics, including avoparcin, bacitracin, spiramycin, tylosin, and virginiamycin [57]. At the same time, the apparent benefits may not be realized. Data have demonstrated that these bans have had negative effects on animal health, which leads to an increase in use of therapeutic antibiotics in some cases [57].

Ultimately, issues affecting the spread of antibiotic resistance among humans, animals, and the environment reflect the need for a One Health approach. One Health has been defined by the WHO as “an approach to designing and implementing programmes, policies, legislation and research in which multiple sectors communicate and work together to achieve better public health outcomes” [58]. Specific areas, such as food safety, zoonotic control, and antimicrobial resistance, are relevant to the One Health approach [58]. The Food and Agriculture Organization of the United Nations has developed policies and proposals to promote action to conserve natural resources, prevent diseases that spread between humans and animals, combat antimicrobial resistance, and protect food safety [59]. As a powerful push for policy in this area, the United Nations included One Health issues in its 2030 Agenda for Sustainable Development, recognizing that a critical issue such as antimicrobial resistance encompasses improvement in human health, animal health, and the environment [60]. The complexity and dynamic nature of the issues involved in the spread of resistance between humans, animals, and the environment will continue to require aggressive policy and effort.

## 6. The Importance of Education Related to Antimicrobial Resistance

To encourage further development of policy targeted to antimicrobial resistance in a sustainable way, medical education is a key target. There are examples of curriculum development in this space. The Infectious Diseases Society of America has created a core curriculum for education around antimicrobial stewardship, and this has been well received by Infectious Diseases fellowship programs [61]. Specifically, a pre- and post-implementation analysis of the curriculum revealed an increase in fellows’ confidence and a significant increase in knowledge of antimicrobial stewardship content areas compared to before the implementation of the curriculum [61].

At the same time, clinical practice patterns are difficult to change [62], and undergraduate medical education is an important area to provide educational initiatives regarding antimicrobial stewardship and resistance. A mapping review evaluated 48 articles related to educational interventions and antimicrobial education for medical trainees. The review demonstrated that while medical schools were including antimicrobial stewardship education in the curriculum, strict evaluation for effectiveness was lacking, and measurement of prescribing behavior after graduation was absent [63]. In a specific example, the Antimicrobial Stewardship, Safety, Utilization, Resistance, and Evaluation (ASSURE) elective was implemented at the Perelman School of Medicine, University of Pennsylvania, to engage medical students in multiple areas of antimicrobial stewardship with a targeted focus on three Ps (Place, Pathogen, Patient), three Ds (Drug, Dose, Duration), and three Cs (Context, Communication, Collaboration). The medical students demonstrated increased confidence in antimicrobial prescribing, fundamental concepts in antimicrobial stewardship, and working as interprofessional teams based on pre- and post-course surveys [64].

In addition to specific curriculum, the incorporation of interprofessional collaboration and leadership into educational interventions to teach concepts related to antimicrobial stewardship and antimicrobial resistance is vital. Simulation has been utilized in both postgraduate and undergraduate settings to enhance understanding of key concepts in antimicrobial stewardship as well as training regarding interprofessional collaboration [65,66]. As an example, an infectious diseases fellowship program designed a simulation series to focus on the quality improvement and population aspects of antimicrobial stewardship [65]. Multidisciplinary roles were created for the simulations, and all fellows participated in the series with moderation led by faculty. At the conclusion of the program, feedback revealed that the faculty enjoyed the program and felt the exercises identified knowledge gaps, while the fellows felt the program was engaging and gave them increased confidence to participate in an ASP [65]. Future efforts should incorporate education regarding national and international policy related to antimicrobial resistance conducted through utilization of simulation and case-based modules.

Although medical education is a vital target for instruction on antimicrobial resistance, veterinary and agriculture sectors are additional targets. A multi-country knowledge, attitudes, and perceptions survey of future veterinary prescribers revealed that only 50.7% of respondents thought antimicrobial resistance was a global human and animal health threat, 50.1% of respondents thought that misuse of antimicrobials by veterinarians contributes significantly to resistance, and 51.5% of respondents thought that inappropriate use of antimicrobials by farmers contributes significantly to antimicrobial resistance [67]. In another study, a comprehensive survey of Australian veterinary students in the last two years of their training was conducted. A total of 476 (38%) out of 1246 veterinary students graduating in either 2017 or 2018 completed the survey, and the results revealed that 88% of respondents felt that the use of antimicrobials by veterinarians had at least a moderate impact on antimicrobial resistance with identification of overuse of antimicrobials in food-producing animals as a significant contributor to antimicrobial resistance [68]. Global educational efforts are needed to continue to shed light on the significance of antimicrobial overuse in animals and the environment.

Finally, the way in which learners receive education is an important consideration. A survey of the membership of the Society for Healthcare Epidemiology of America demonstrated that continuing medical education subject matter was the most important factor affecting media preference, while local grand rounds and regional meetings were the most preferred media [69]. In contemporary medical education, digital methods and social media have received increasing attention as an educational strategy. Debra Goff and colleagues have previously discussed the potential impact of increased use of social media for infectious diseases education, noting that a Pubmed search of “Twitter medical education” included 56 unique articles. In addition, the authors noted that journal clubs conducted through social media have greater international reach, and content from scientific meetings can be amplified, but at the same time, ensuring patient confidentiality is a concern [70]. Future educational efforts will undoubtedly utilize digital and social media strategies, and this can be leveraged to promote policy for preventing the spread of antimicrobial resistance, including educational instruction regarding One Health issues.

## 7. Future Directions for Policy and Education to Combat Antimicrobial Resistance

Comprehensive policy and education focused on the specific areas of antimicrobial stewardship, drug development, and environmental, agricultural, and One Health initiatives are critical to combat the threat of antimicrobial resistance (Table 2). These issues all coalesce to affect humans, animals, and the environment. Combating antimicrobial resistance cannot be done with a silo mentality, and further collaborative efforts are needed in this field.

Continued guidance and regulation around mandates reinforcing the importance of antimicrobial stewardship is vital. Future efforts should require increased reporting of antimicrobial usage data and the effect of ASPs on clinical outcomes, such as mortality, length of stay, and resistance rates. More statewide collaboratives should be encouraged to share best practices with the goal of significantly improving patient safety and quality.

Future reforms should also be aimed at incentivization for collaborations in the pharmaceutical industry to develop agents targeted to antimicrobial resistance. Specifically, investment in smaller companies interested in early-stage drug development is crucial. In addition to drug development, future funding and support should be dedicated to innovative technology and therapeutics, such as diagnostic testing, vaccination, and phage therapy.

Since humans, animals, and the environment are all connected, future policy, support, and economic resources should be aimed at strengthening One Health programs. Antimicrobial use and resistance should not only be monitored in humans but also in animal settings and the environment.

Finally, educational curriculum development for trainees will be critical in the fight to combat antimicrobial resistance. Current examples of curriculum development focus on fundamentals of antimicrobial stewardship, but education around issues related to policy development and leadership in this field are an unmet need. Additionally, the development of curriculum to educate on the One Health approach to combating antimicrobial resistance would be welcome. Only through education and policies enacted to support these complex issues will combating antimicrobial resistance be fully realized.

## Figures and Tables

**Table 1:** The CDC Core Elements of Hospital Antibiotic Stewardship Programs.

Core Element	Summary
Leadership commitment	Dedication of necessary resources (i.e., human, financial, information technology) Hospital leadership commitment (i.e., dedicated time for ASP leadership)
Accountability	Appointment of a leader or co-leaders to have responsibility for program management and outcomes Physician and pharmacy co-leadership ideal and effective
Pharmacy expertise	Appointment of pharmacist to lead implementation efforts for the ASP Pharmacy engagement is critical
Action	Implementation of ASP interventions (i.e., prospective audit and feedback, preauthorization) Specific focus on actions related to CAP, UTI, SSTI
Tracking	Monitoring of antimicrobial prescribing and outcomes (i.e., CDI, resistance patterns) Development of priority process measures to assess the impact of interventions
Reporting	Reporting antimicrobial usage and resistance patterns to healthcare staff (i.e., providers, physicians, pharmacists, nursing, hospital administration)
Education	Education devoted to appropriate antimicrobial prescribing, adverse reactions due to antimicrobials, and antimicrobial resistance Case-based education is particularly effective in providing targeted education Nursing education important

Abbreviations: CDC—Centers for Disease Control and Prevention; ASP—antimicrobial stewardship program; CAP—community-acquired pneumonia; UTI—urinary tract infection; SSTI—skin and soft tissue infection; CDI—*Clostridioides difficile* infection.

**Table 2:** Recommendations for comprehensive policy and education.

Area of Focus	Recommendations
Antimicrobial stewardship	Continued mandates with reporting of key outcomes related to antimicrobial usage and ASP interventions Development of regulations regarding FTE allocation for leadership of the ASP Further development of statewide collaboratives Increased educational interventions such as simulation to engage learners in critical aspects of antimicrobial stewardship, resistance, and policy
Drug development	Pass legislation currently in US Congress (i.e., Disarm Act, Pasteur Act) Develop policy to support smaller pharmaceutical companies interested in antimicrobial development Investment in further research of new diagnostics, vaccines, and therapeutics designed to detect, prevent, and treat resistance
Environment/Agriculture/One Health	Continued development of policy designed to monitor antimicrobial resistance in animal settings and the environment Further collaborative efforts to form and strengthen One Health programs Incorporation of education around environmental and One Health policy and programs to prevent the spread of resistance

Abbreviations: ASP—antimicrobial stewardship program; FTE—full-time equivalent; US—United States.

## Data Availability

Not applicable.
